# Association of Antidementia Therapies With Time to Skilled Nursing Facility Admission and Cardiovascular Events Among Elderly Adults With Alzheimer Disease

**DOI:** 10.1001/jamanetworkopen.2019.0213

**Published:** 2019-03-01

**Authors:** Alvaro San-Juan-Rodriguez, Yuting Zhang, Meiqi He, Inmaculada Hernandez

**Affiliations:** 1 Department of Pharmacy and Therapeutics, School of Pharmacy, University of Pittsburgh, Pittsburgh, Pennsylvania; 2 Melbourne Institute, Faculty of Business and Economics, University of Melbourne, Melbourne, Victoria, Australia

## Abstract

**Importance:**

To date, no study has compared time to skilled nursing facility (SNF) admission and cardiovascular events across medications available to treat Alzheimer disease.

**Objective:**

To compare time to SNF admission and cardiovascular events between acetylcholinesterase inhibitor (AChEI) monotherapy, memantine hydrochloride monotherapy, and combination therapy with an AChEI and memantine in treating elderly adults with Alzheimer disease.

**Design, Setting, and Participants:**

This retrospective cohort study uses January 1, 2006, to December 31, 2014, claims data from a 5% random sample of Medicare beneficiaries who had received a new diagnosis of Alzheimer disease between January 1, 2007, and December 31, 2013, and who initiated AChEI monotherapy, memantine monotherapy, or combination therapy with an AChEI and memantine (N = 73 475). Patients were followed up until discontinuation of treatment, switch of treatment, death, or the end of the study period. Statistical analysis was conducted from February 15, 2018, to June 15, 2018.

**Exposures:**

Acetylcholinesterase inhibitor monotherapy (n = 44 424), memantine monotherapy (n = 11 809), and combination therapy with an AChEI and memantine (n = 17 242).

**Main Outcomes and Measures:**

Primary outcomes were time to SNF admission and the composite of the following cardiovascular events: acute myocardial infarction, bradycardia, syncope, atrioventricular block, QT interval prolongation, and ventricular tachycardia. Cox proportional hazards regression models were constructed to compare outcomes between each pair of treatment groups, controlling for a comprehensive list of patient characteristics.

**Results:**

The study population included 73 475 participants (53 068 women and 20 407 men; mean [SD] age, 81.8 [8.3] years); 25.5% of the participants initiating AChEI monotherapy, 25.6% of participants initiating memantine monotherapy, and 29.7% of participants initiating combination therapy with an AChEI and memantine were admitted to an SNF. Similarly, 22.2% of the participants initiating AChEI monotherapy, 20.0% of those initiating memantine monotherapy, and 24.5% of those initiating combination therapy experienced at least 1 cardiovascular event. No difference in time to SNF admission was found across the 3 treatment groups. The risk of the composite measure of any cardiovascular event did not differ between the combination therapy and AChEI monotherapy groups (adjusted hazard ratio [aHR], 0.99; 95% CI, 0.96-1.03); however, it was higher for both AChEI monotherapy (aHR, 1.07; 95% CI, 1.02-1.12) and combination therapy (aHR, 1.07; 95% CI, 1.01-1.12), relative to memantine monotherapy. This result was mainly driven by the lower risk of bradycardia and syncope observed for the memantine monotherapy group relative to both AChEI monotherapy (bradycardia: aHR, 0.88; 95% CI, 0.82-0.95; and syncope: aHR, 0.92; 95% CI, 0.86-0.97) and combination therapy (bradycardia: aHR, 0.89; 95% CI, 0.82-0.97; and syncope: aHR, 0.87; 95% CI, 0.83-0.94).

**Conclusions and Relevance:**

Time to SNF admission did not differ across treatment groups, but memantine monotherapy was associated with a lower risk of cardiovascular events compared with both AChEI monotherapy and combination therapy with an AChEI and memantine.

## Key Points

**Question:**

How do time to skilled nursing facility admission and cardiovascular events compare between acetylcholinesterase inhibitor monotherapy, memantine monotherapy, and combination therapy with an acetylcholinesterase inhibitor and memantine in treating Alzheimer disease?

**Findings:**

In this cohort study using 2006-2014 Medicare data from 73 475 patients with a new diagnosis of Alzheimer disease, no differences were found in time to skilled nursing facility admission across treatments. Memantine monotherapy was associated with a 7% lower risk of any cardiovascular event compared with both acetylcholinesterase inhibitor monotherapy and combination therapy.

**Meaning:**

No differences in time to skilled nursing facility admission were found across treatments; however, memantine was associated with a lower risk of cardiovascular events.

## Teaser

This cohort study uses Medicare data to compare time to skilled nursing facility admission and cardiovascular (CV) events among patients with Alzheimer disease receiving acetylcholinesterase inhibitor monotherapy, memantine monotherapy, or combination acetylcholinesterase inhibitor and memantine therapy.

## Introduction

Alzheimer disease (AD) is the most prevalent cause of dementia. It is characterized by an insidious deterioration of cognitive functions and motor skills, with distinctive behavioral and psychological manifestations.^1^ Recent estimates suggest that 5.7 million people in the United States currently have AD,^2^ and as the population ages, the prevalence of AD will drastically increase.^3^

There are 4 antidementia drugs approved by the US Food and Drug Administration to treat AD, including 3 acetylcholinesterase inhibitors (AChEIs)—donepezil hydrochloride, rivastigmine tartrate, and galantamine hydrobromide—and the *N*-methyl-d-aspartic receptor antagonist memantine hydrochloride.^2^ Rivastigmine and galantamine were approved for mild to moderate AD, donepezil for all stages of AD, and memantine for moderate to severe AD.^4,5,6,7,8,9^ In addition, combination therapy with an AChEI and memantine was approved for moderate to severe AD, based on evidence from randomized clinical trials showing modestly superior cognitive, behavioral, and psychological performance with combination therapy with donepezil and memantine compared with donepezil alone.^10,11,12,13,14,15,16^

All 4 antidementia drugs have been associated with an increased incidence of adverse cardiovascular events.^17^ Specifically, AChEIs have been associated with an increased risk of bradycardia^18,19,20,21^ and syncope^8,22^ but also with QT interval prolongation, ventricular tachycardia, and atrioventricular block.^23,24,25,26^ In addition, memantine has been associated with an increased risk of acute myocardial infarction (AMI),^27^ among other adverse cardiovascular events.^28^ Despite the severity of some of these events, only 1 study has compared their incidence across the 4 different antidementia monotherapies. Using data from a Danish registry, Fosbøl et al^27^ found that all 3 AChEIs were comparable but that memantine was associated with an increased risk of AMI. However, the results from this study were strongly affected by selection bias because patients in the memantine group were considerably sicker than those receiving AChEIs.^17^ To our knowledge, no study has evaluated whether combination therapy with AChEIs and memantine is associated with a higher risk of adverse cardiovascular events relative to monotherapy.

In this study, we used 2006-2014 claims data from Medicare Part D beneficiaries with a new diagnosis of AD and compared time to skilled nursing facility (SNF) admission and time to cardiovascular events with AChEI monotherapy, memantine monotherapy, and combination therapy with an AChEI and memantine. We further compared the incidence of these outcomes across 5 treatment groups defined on the basis of the antidementia treatment that patients initiated (donepezil monotherapy, rivastigmine monotherapy, galantamine monotherapy, memantine monotherapy, or combination therapy with an AChEI and memantine).

## Methods

### Data Source and Study Population

**Figure 1.**
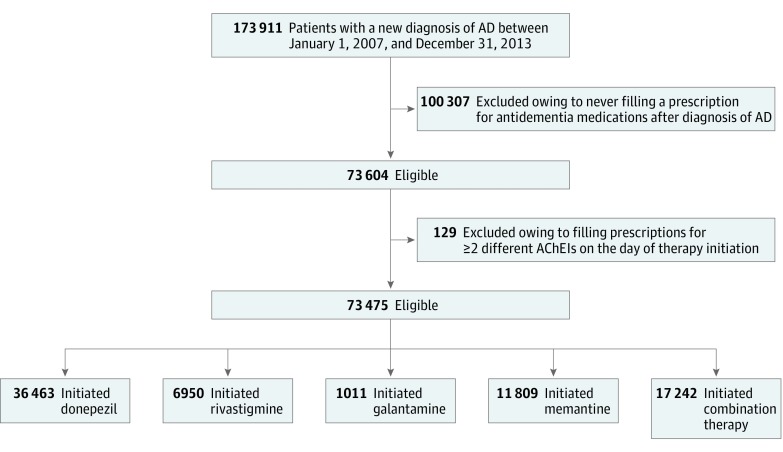
Selection of the Study Sample In selecting the study sample, Medicare Part D beneficiaries who had a new diagnosis of Alzheimer disease (AD) between 2007 and 2013 and had filled at least 1 prescription for an antidementia drug after diagnosis of AD were identified. Those who filled prescriptions for 2 different acetylcholinesterase inhibitors (AChEIs) on the day of therapy initiation were excluded.

We obtained medical and pharmacy claims data from January 1, 2006, to December 31, 2014, for a 5% random sample of Medicare beneficiaries from the Centers for Medicare & Medicaid Services (CMS). First, we identified all patients with a new diagnosis of AD between January 1, 2007, and December 31, 2013 (N = 173 911) (Figure 1). We used the CMS Chronic Conditions Data Warehouse definition of AD, which defines AD as having at least 1 claim with *International Classification of Diseases, Ninth Revision* (*ICD-9*) code 331.0.^29^ Second, we collected the prescriptions filled for antidementia drugs, including AChEIs (donepezil, rivastigmine, and galantamine) and memantine between the day of the first AD diagnosis and the end of the study period. The day of the first prescription filled for an antidementia drug was defined as the day of therapy initiation. We excluded patients who did not fill prescriptions for any of the antidementia medications mentioned (n = 100 307) or who filled prescriptions for 2 different AChEIs on the day of therapy initiation (n = 129). Third, using prescription fill dates and days of supply, we calculated the number of days that a patient had a concurrent supply of an AChEI and memantine in the first 60 days after therapy initiation. Then, we categorized our study sample in the following 2 groups: monotherapy, which included patients who had less than 14 days with concurrent AChEI and memantine use in the first 60 days after therapy initiation, and combination therapy, which included those with 14 or more days with an overlapping supply of both medication classes in this time window.^30^ We further divided the monotherapy group on the basis of the specific antidementia drug initiated. For the monotherapy treatment groups, the index date was defined as the date of the first prescription for an antidementia drug (therapy initiation date); for the combination therapy treatment group, the index date was defined as the first day with an overlapping supply of AChEIs and memantine. All patients were followed up from the index date until the first of the following events: switch to a treatment group different from the original, discontinuation of treatment (defined as not having a treatment supply for ≥60 days), death, or the end of the study period. Specifically, patients in monotherapy treatment groups (AChEI or memantine monotherapy) were censored when they filled a prescription for an antidementia agent other than the one originally initiated (switch to other monotherapy group or to combination therapy) or when they had 60 days without a treatment supply (discontinuation). Patients in the combination therapy group were censored when they had a gap greater than 60 days without concurrent use of an AChEI and memantine (this would represent complete discontinuation of the combination therapy or a switch to a monotherapy treatment group). This study followed the Strengthening the Reporting of Observational Studies in Epidemiology (STROBE) reporting guideline for cohort studies.^31^ This study was approved by the Institutional Review Board at the University of Pittsburgh as exempt owing to the use of unidentifiable data.

### Outcomes

Our primary outcomes were time to SNF admission and time to occurrence of any adverse cardiovascular event, which was defined as the composite measure of 1 of the following: AMI (*ICD-9* code 410), bradycardia (*ICD-9* code 427.89), syncope (*ICD-9* code 780.2), atrioventricular block (*ICD-9* code 426.0), QT interval prolongation (*ICD-9* code 426.82), and ventricular tachycardia (*ICD-9* code 427.1). All of these cardiovascular events had been previously described as potential adverse cardiovascular events of antidementia medications.^8,17,18,19,20,21,22,23,24,25,26,27,28^ Secondary outcomes included the occurrence of AMI, bradycardia, syncope, atrioventricular block, QT interval prolongation, or ventricular tachycardia. Time to SNF admission was defined using the short stay/long stay/SNF variable captured in the Medicare Provider Analysis and Review data file. To define cardiovascular outcomes, we collected all inpatient and outpatient claims during the follow-up period with the *ICD-9* codes already specified.

### Covariates

Baseline characteristics included demographic characteristics, clinical characteristics, and a history of SNF admission in the year before the index date. The demographic characteristics included age, sex, race/ethnicity, and eligibility for Medicare coverage due to disability. The clinical characteristics included a history of AMI, bradycardia, syncope, atrioventricular block, QT interval prolongation, and ventricular tachycardia before the index date and each of the 25 CMS priority conditions (all 27 CMS priority conditions except for AD and AD or other dementia).^29^ In defining a history of any of the cardiovascular events of interest, we used the *ICD-9* codes specified in the year before the index date; in defining CMS priority conditions, we used the CMS Chronic Condition Data Warehouse indicators that trace back the first diagnosis of these conditions to 1999.^29^

### Statistical Analysis

Statistical analysis was conducted from February 15, 2018, to June 15, 2018. We compared baseline patient characteristics across treatment groups using analysis of variance for continuous variables and the χ^2^ test for categorical variables. We constructed Kaplan-Meier curves to estimate the unadjusted incidence rates of the primary outcomes at 1, 2, and 3 years of follow-up. To further compare how outcomes differed across treatment groups, while controlling for differences in patient characteristics, we used Cox proportional hazards regression models adjusted for all the covariates listed. Specifically, we first constructed Cox proportional hazards regression models to compare outcomes across the AChEI monotherapy, memantine monotherapy, and combination therapy groups. In doing so, Cox proportional hazards regression models included indicator variables for memantine monotherapy and combination therapy (AChEI monotherapy was the omitted reference group). Then, head-to-head comparisons between the different treatment groups were calculated as the ratios of 2 hazard ratios. To mitigate the increased chance of type I error associated with the performance of pairwise comparisons, we used the Bonferroni correction and set the level of individual significance at *P* < .016 (0.05/3). We further compared the outcomes across 5 treatment groups defined on the basis of the antidementia drug initiated (donepezil monotherapy, rivastigmine monotherapy, galantamine monotherapy, memantine monotherapy, or combination therapy). In doing so, we followed the same method, but the level of individual significance was set at *P* < .005 (0.05/10) because of the 10 possible pairwise comparisons between treatment groups. For all time-to-event analyses, time 0 was the index date, and the time at risk was censored at the switch of treatment groups, the discontinuation of treatment, death, or the end of the study period. All analyses were conducted with SAS, version 9.4 (SAS Institute Inc).

## Results

### Patient Characteristics

Our study population included 73 475 participants (53 068 women and 20 407 men; mean [SD] age, 81.8 [8.3] years) (Table 1).^29^ The sample included 36 463 participants (49.6%) who initiated monotherapy with donepezil, 6950 (9.5%) who initiated monotherapy with rivastigmine, 1011 (1.4%) who initiated monotherapy with galantamine, 11 809 (16.1%) who initiated monotherapy with memantine, and 17 242 (23.5%) who initiated AChEI and memantine combination therapy. The baseline age was the highest for the memantine monotherapy treatment group (mean [SD], 82.3 [8.3] years) and lowest for the combination therapy group (mean [SD], 81.4 [8.2] years). The galantamine group had the highest proportion of men (35.1%), whereas the donepezil group had the lowest (27.0%). The donepezil group had the most racially diverse composition, with 81.0% of patients identified as white, whereas the galantamine group had the least racially diverse composition, with 87.4% of patients identified as white. The galantamine treatment group had the lowest proportion of patients admitted to an SNF in the year before the index date (20.3%), as well as the lowest incidence of cardiovascular events (such as history of atrioventricular block [0.6%], AMI [2.3%], QT interval prolongation [0%], or syncope [10.3%]) in the year before the index date. The mean (SD) follow-up period was 1.26 (1.31) years for patients receiving AChEI monotherapy (1.29 [1.33] years for donepezil, 1.11 [1.22] years for rivastigmine, and 1.30 [1.22] years for galantamine), 1.21 (1.29) years for the memantine monotherapy group, and 1.54 (1.44) years for the combination therapy group.

**Table 1.** Baseline Characteristics of Patients by Treatment Group

Variable	Patients, No. (%)						*P* Value^a^
	Overall (N = 73 475)	Donepezil (n = 36 463)	Galantamine (n = 1011)	Rivastigmine (n = 6950)	Memantine (n = 11 809)	Combination Therapy (n = 17 242)	
Age, mean (SD), y	81.8 (8.3)	81.9 (8.3)	81.8 (8.1)	81.8 (8.3)	82.3 (8.3)	81.4 (8.2)	<.001
Male sex	20 407 (27.8)	9825 (27.0)	355 (35.1)	1915 (27.6)	3261 (27.6)	5051 (29.3)	<.001
Race/ethnicity							
White	60 592 (82.5)	29 540 (81.0)	884 (87.4)	5711 (82.2)	9821 (83.2)	14 636 (84.9)	<.001
Black	7626 (10.4)	4172 (11.4)	82 (8.1)	658 (9.5)	1104 (9.4)	1610 (9.3)	
Hispanic	2780 (3.8)	1433 (3.9)	27 (2.7)	271 (3.9)	500 (4.2)	549 (3.2)	
Other	2477 (3.4)	1318 (3.6)	18 (1.8)	310 (4.5)	384 (3.3)	447 (2.6)	
Disabled	2065 (2.8)	1007 (2.8)	25 (2.5)	183 (2.6)	325 (2.8)	525 (3.0)	.27
History of SNF admission	19 744 (26.9)	9676 (26.5)	205 (20.3)	1966 (28.3)	3235 (27.4)	4662 (27.0)	<.001
History of selected cardiovascular events^b^							
Atrioventricular block	691 (0.9)	344 (0.9)	6 (0.6)	72 (1.0)	122 (1.0)	147 (0.9)	.34
Acute myocardial infarction	1954 (2.7)	1054 (2.9)	23 (2.3)	180 (2.6)	300 (2.5)	397 (2.3)	.002
Bradycardia	4934 (6.7)	2442 (6.7)	68 (6.7)	461 (6.6)	835 (7.1)	1128 (6.5)	.51
QT interval prolongation	43 (0.1)	27 (0.1)	0	1 (0.01)	7 (0.1)	8 (0.1)	.30
Syncope	9249 (12.6)	4593 (12.6)	104 (10.3)	899 (12.9)	1538 (13.0)	2115 (12.3)	.06
Ventricular tachycardia	807 (1.1)	430 (1.2)	15 (1.5)	86 (1.2)	118 (1.0)	158 (0.9)	.03
CMS priority conditions^c^							
Acquired hypothyroidism	13 672 (18.6)	6591 (18.1)	308 (30.5)	1428 (20.6)	2213 (18.7)	3132 (18.2)	<.001
Acute myocardial infarction	5130 (7.0)	2638 (7.2)	62 (6.1)	476 (6.9)	848 (7.2)	1106 (6.4)	.007
Anemia	33 022 (44.9)	15 887 (43.6)	698 (69.0)	3456 (49.7)	5360 (45.4)	7621 (44.2)	<.001
Asthma	6749 (9.2)	3325 (9.1)	149 (14.7)	754 (10.9)	1099 (9.3)	1422 (8.3)	<.001
Atrial fibrillation	15 792 (21.5)	8022 (22.0)	208 (20.6)	1569 (22.6)	2590 (21.9)	3403 (19.7)	<.001
Benign prostatic hyperplasia	7508 (10.2)	3463 (9.5)	197 (19.5)	780 (11.2)	1226 (10.4)	1842 (10.7)	<.001
Cataract	54 960 (74.8)	27 290 (74.8)	792 (78.3)	5368 (77.2)	8827 (74.8)	12 683 (73.6)	<.001
COPD	25 609 (34.9)	12 890 (35.4)	334 (33.0)	2594 (37.3)	4197 (35.5)	5594 (32.4)	<.001
Chronic kidney disease	24 712 (33.6)	12 442 (34.1)	325 (32.2)	2475 (35.6)	4086 (34.6)	5384 (31.2)	<.001
Congestive heart failure	33 409 (45.5)	16 901 (46.4)	418 (41.4)	3291 (47.4)	5498 (46.6)	7301 (42.3)	<.001
Depression	42 885 (58.4)	20 708 (56.8)	600 (59.4)	4227 (60.8)	7040 (59.6)	10 310 (59.8)	<.001
Diabetes	31 134 (42.4)	15 693 (43.0)	425 (42.0)	3098 (44.6)	5057 (42.8)	6861 (39.8)	<.001
Glaucoma	19 183 (26.1)	9607 (26.4)	279 (27.6)	1875 (27.0)	3127 (26.5)	4295 (24.9)	<.001
Hip or knee fracture	8231 (11.2)	4002 (11.0)	103 (10.2)	853 (12.3)	1407 (11.9)	1866 (10.8)	<.001
History of stroke or TIA	23 985 (32.6)	11 808 (32.4)	336 (33.2)	2489 (35.8)	3930 (33.3)	5422 (31.5)	<.001
Hypertension	40 430 (55.0)	19 516 (53.5)	877 (86.8)	4174 (60.1)	6446 (54.6)	9417 (54.6)	<.001
Hyperlipidemia	36 110 (49.1)	17 477 (47.9)	817 (80.8)	3709 (53.4)	5748 (48.7)	8359 (48.5)	<.001
Ischemic heart disease	47 050 (64.0)	23 619 (64.8)	651 (64.4)	4661 (67.1)	7606 (64.4)	10 513 (61.0)	<.001
Osteoporosis	30 470 (41.5)	15 188 (41.7)	360 (35.6)	3089 (44.5)	4982 (42.2)	6851 (39.7)	<.001
Rheumatoid arthritis or osteoarthritis	47 283 (64.4)	23 691 (65.0)	686 (67.9)	4655 (67.0)	7687 (65.1)	10 564 (61.3)	<.001
History of cancer							
Breast	4508 (6.1)	2269 (6.2)	64 (6.3)	436 (6.3)	745 (6.3)	994 (5.8)	.24
Colorectal	2590 (3.5)	1275 (3.5)	35 (3.5)	264 (3.8)	449 (3.8)	567 (3.3)	.13
Prostate	3499 (4.8)	1722 (4.7)	58 (5.7)	326 (4.7)	540 (4.6)	853 (5.0)	.34
Lung	1160 (1.6)	600 (1.7)	19 (1.9)	113 (1.6)	184 (1.6)	244 (1.4)	.33
Endometrial	639 (0.9)	315 (0.9)	7 (0.7)	77 (1.1)	103 (0.9)	137 (0.8)	.19

Abbreviations: CMS, Centers for Medicare & Medicaid Services; COPD, chronic obstructive pulmonary disease; SNF, skilled nursing facility; TIA, transient ischemic attack.

^a^ To test the existence of any significant difference across all treatment groups (ie, donepezil, galantamine, rivastigmine, memantine, and combination therapy).

^b^ Variables for history of cardiovascular events were defined using the *International Classification of Diseases, Ninth Revision* codes listed in the Outcomes subsection of the Methods section and the claims in the year before the index date.

^c^ All CMS priority chronic conditions were defined using the CMS Chronic Conditions Data Warehouse definitions of these conditions, which track the first diagnosis back to January 1999.^29^

### Unadjusted Incidence of SNF Admissions and Cardiovascular Events

Table 2 shows the number of outcome events across treatment groups. A total of 25.5% of the participants initiating AChEI monotherapy, 25.6% of those initiating memantine monotherapy, and 29.7% of those initiating combination AChEI and memantine were admitted to an SNF. Similarly, 22.2% of the participants initiating AChEI monotherapy, 20.0% of those initiating memantine monotherapy, and 24.5% of those initiating combination AChEI and memantine experienced at least 1 cardiovascular event. eTable 1 in the Supplement shows the unadjusted incidence rates at years 1, 2, and 3 obtained from Kaplan-Meier curves. There were no differences in the unadjusted incidence of SNF admission across treatment groups. For instance, at year 1 of follow-up, the unadjusted incidence of SNF admission was 0.209 (95% CI, 0.204-0.214) for patients receiving donepezil, 0.189 (95% CI, 0.161-0.217) for patients receiving galantamine, 0.213 (95% CI, 0.201-0.224) for patients receiving rivastigmine, 0.220 (95% CI, 0.212-0.229) for patients receiving memantine, and 0.204 (95% CI, 0.197-0.211) for patients receiving combination therapy. At year 1 of follow-up, the unadjusted incidence of any adverse cardiovascular event was lower for memantine (0.18; 95% CI, 0.17-0.18) and combination therapy (0.18; 95% CI, 0.17-0.18) than for donepezil monotherapy (0.19; 95% CI, 0.19-0.20).

**Table 2.** Data on Number and Frequency of Outcome Events by Treatment Group

Event	No. of Events (%)					*P* Value
	Donepezil (n = 36 463)	Galantamine (n = 1011)	Rivastigmine (n = 6950)	Memantine (n = 11 809)	Combination therapy (n = 17 242)	
Skilled nursing facility admission	9476 (26.0)	234 (23.2)	1609 (23.2)	3022 (25.6)	5112 (29.7)	<.001
Any cardiovascular adverse event	8268 (22.7)	223 (22.1)	1352 (19.5)	2365 (20.0)	4215 (24.5)	<.001
Acute myocardial infarction	1509 (4.1)	33 (3.3)	255 (3.7)	442 (3.7)	665 (3.9)	.01
Atrioventricular block	450 (1.2)	6 (0.6)	64 (0.9)	119 (1.0)	174 (1.0)	.01
Bradycardia	3325 (9.1)	93 (9.2)	503 (7.2)	885 (7.5)	1697 (9.8)	<.001
QT interval prolongation	41 (0.1)	2 (0.2)	1 (0.01)	16 (0.1)	19 (0.1)	.12
Syncope	4715 (12.9)	130 (12.9)	750 (10.8)	1306 (11.1)	2514 (14.6)	<.001
Ventricular tachycardia	479 (1.3)	14 (1.4)	93 (1.3)	144 (1.2)	215 (1.3)	.90

### Adjusted Hazard Ratios of SNF Admissions and Cardiovascular Events

#### Comparison Between AChEI Monotherapy, Memantine Monotherapy, and Combination Therapy

**Figure 2.**
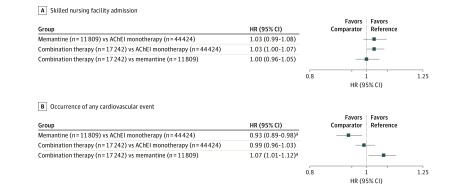
Adjusted Hazard Ratios (HRs) for Primary Outcomes for the Comparison Between Acetylcholinesterase Inhibitor (AChEI) Monotherapy, Memantine Monotherapy, and Combination Therapy The comparator group is the first item and the reference group is the second item. Adjusted HRs were obtained from Cox proportional hazards regression models that controlled for age, sex, race/ethnicity, disability, history of acute myocardial infarction, history of bradycardia, history of syncope, history of atrioventricular block, history of QT interval prolongation, history of ventricular tachycardia, and each of 25 Centers for Medicare & Medicaid Services priority conditions (all 27 Centers for Medicare & Medicaid Services priority conditions except for Alzheimer disease and Alzheimer disease or other dementia).^29^ ^a^Bonferroni-corrected *P* < .016.

Figure 2 shows the adjusted hazard ratios (aHRs) for primary outcomes for the 3 pairwise comparisons between AChEI monotherapy, memantine monotherapy, and combination therapy^29^; eTable 2 in the Supplement shows the aHRs for secondary outcomes. Time to SNF admission did not differ between treatment groups (Figure 2).^29^ Acetylcholinesterase inhibitor monotherapy and combination therapy were both associated with a higher risk of any cardiovascular event compared with memantine monotherapy. Specifically, the aHR for any cardiovascular event was 1.07 (95% CI, 1.02-1.12) for the AChEI monotherapy group and 1.07 (95% CI, 1.01-1.12) for the combination therapy group, both relative to the memantine monotherapy group. The risk of any cardiovascular event did not differ between the combination therapy and AChEI monotherapy groups (aHR, 0.99; 95% CI, 0.96-1.03).

The observed differences for the composite outcome of any cardiovascular event were mostly driven by differences in the outcomes of bradycardia and syncope (eTable 2 in the Supplement). In particular, the memantine monotherapy group was associated with a lower risk of bradycardia and syncope relative to both AChEI monotherapy (bradycardia: aHR, 0.88; 95% CI, 0.82-0.95; and syncope: aHR, 0.92; 95% CI, 0.86-0.97) and combination therapy (bradycardia: aHR, 0.89; 95% CI, 0.82-0.97; and syncope: aHR, 0.87; 95% CI, 0.83-0.94). The risk of other cardiovascular events, including AMI, atrioventricular block, QT interval prolongation, and ventricular tachycardia, did not differ between AChEI monotherapy and memantine monotherapy, and between combination therapy and memantine monotherapy.

#### Multiple Comparisons Between 5 Treatment Groups

**Figure 3.**
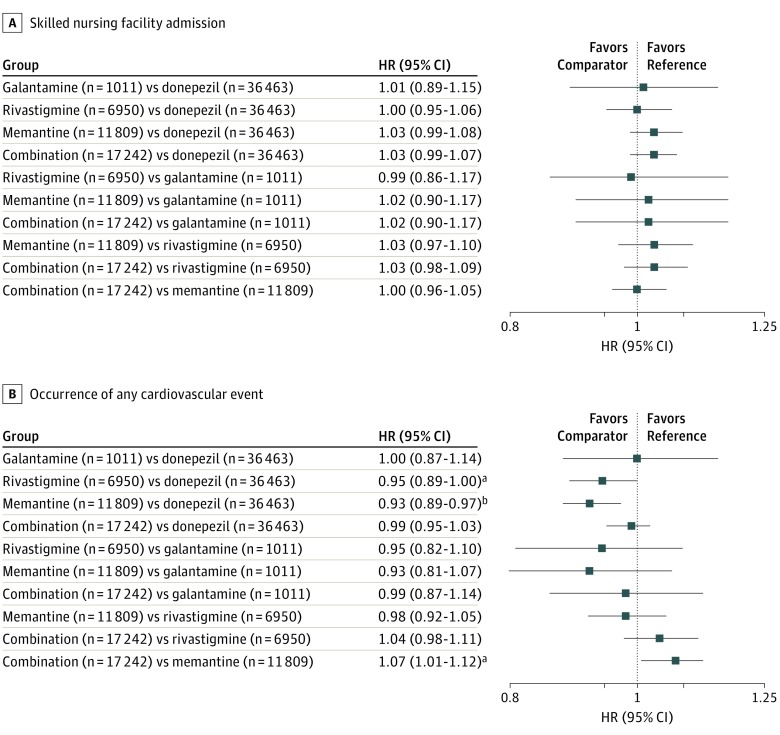
Adjusted Hazard Ratios (HRs) for Primary Outcomes, by Treatment Groups The comparator group is the first item and the reference group is the second item. Adjusted HRs were obtained from Cox proportional hazards regression models that controlled for age, sex, race/ethnicity, disability, history of acute myocardial infarction, history of bradycardia, history of syncope, history of atrioventricular block, history of QT interval prolongation, history of ventricular tachycardia, and each of 25 Centers for Medicare & Medicaid Services priority conditions (all 27 Centers for Medicare & Medicaid Services priority conditions except for Alzheimer disease and Alzheimer disease or other dementia).^29^ ^a^*P* < .05. ^b^Bonferroni-corrected *P* < .005.

Figure 3 shows the aHRs for primary outcomes for the 10 pairwise comparisons between 5 treatment groups^29^; eTable 3 in the Supplement shows the aHRs for secondary outcomes. Again, the risk of SNF admission did not differ among treatment groups (Figure 3).^29^ After the application of Bonferroni correction, the only statistically significant difference for the primary outcome of any cardiovascular event was for the comparison between the memantine monotherapy and donepezil monotherapy treatment groups. Specifically, the aHR for any cardiovascular event was 0.93 (95% CI, 0.89-0.97) for memantine compared with donepezil. The differences observed in the incidence of any cardiovascular events between the donepezil and memantine monotherapy groups were again driven by the outcomes of bradycardia and syncope; memantine monotherapy had a decreased risk of bradycardia (aHR, 0.87; 95% CI, 0.81-0.94) and syncope (aHR, 0.91; 95% CI, 0.85-0.96) compared with donepezil (eTable 3 in the Supplement).

## Discussion

To our knowledge, this study is the first to compare time to SNF admission and time to occurrence of adverse cardiovascular events across all therapeutic strategies currently available to treat AD. Our study yielded 3 main findings. First, we found that time to SNF admission did not differ across treatment groups. Second, the aHR of any cardiovascular event was 7% lower for memantine monotherapy relative to both AChEI monotherapy and combination therapy. This result was driven mainly by the lower risk of bradycardia and syncope observed for the memantine monotherapy group. Third, when we compared the occurrence of cardiovascular events across the 5 treatment groups defined by the antidementia treatment initiated, we observed that memantine monotherapy was associated with a lower risk than donepezil monotherapy of any cardiovascular event, bradycardia, and syncope.

We are aware of only 1 previous observational study that compared time to SNF admission across the different antidementia therapies in real-world practice. Lopez et al^32^ used 1997-2004 data from a cohort of 429 patients with AD enrolled in a US registry and found that combination therapy with an AChEI and memantine was associated with a reduced risk of SNF admission compared with AChEI monotherapy. Secondary and post hoc analyses of the DOMINO-AD (Donepezil and Memantine in Moderate to Severe Alzheimer’s Disease) trial,^33^ originally conducted between 2008 and 2010 to compare the effectiveness of placebo, donepezil monotherapy, memantine monotherapy, and combination therapy with donepezil and memantine in patients with AD transitioning from a moderate to a severe stage, did not find significant differences in time to SNF admission. Consistent with that study, our findings suggest that there are no significant differences in time to SNF admission across different antidementia treatment groups. A potential explanation for differences in the results between the study by Lopez et al,^32^ the DOMINO-AD trial,^33^ and our study may be the years captured by each study. The DOMINO-AD trial (2008-2010)^33^ and our study (2007-2014) used more recent data than did Lopez et al (1997-2004),^32^ and nonpharmacologic patterns of care may have changed over time. Specially, multiple alternatives to nursing homes have emerged in recent years, such as in-home health care services, adult day centers, or retirement residences, which try to keep patients with AD in the community instead of in nursing homes.^34^

Using data from a Danish registry and from Medicare, Fosbøl et al^27^ found that the risk of AMI, heart failure, and syncope or atrioventricular block did not differ across AChEI monotherapies (donepezil, galantamine, and rivastigmine). These results are consistent with our observations. In addition, Fosbøl et al^27^ observed a higher risk of AMI and cardiac death with memantine monotherapy in the Danish cohort but not in the Medicare cohort. Our results are consistent with these findings because we did not find differences in the risk of AMI across treatment groups.

Our study has important clinical implications. We found that both AChEI monotherapy and combination therapy with an AChEI and memantine are associated with a 7% higher aHR of any cardiovascular events compared with memantine monotherapy. This higher risk was driven mostly by a higher risk of bradycardia and syncope, which are not as severe or life threatening as other cardiovascular events assessed in this study. Thus, the clinical significance of this increased risk remains uncertain. Future studies should assess clinical, functional, and utilization outcomes of patients with AD who are experiencing bradycardia or syncope to clarify the clinical significance of our observations. In addition, further research should validate these findings in other cohorts of patients, using data that better capture cognitive, behavioral, and psychological function. Leveraging this type of data would enable comparisons of the risk of outcomes between treated and nontreated patients, which would further establish the role of antidementia therapies in the delay of cognitive impairment associated with AD. If differences in the occurrence of cardiovascular events were confirmed, clinicians may start evaluating cardiovascular safety profiles of the different antidementia medications before prescribing a treatment for their patients. Until then, new longitudinal studies that compare the effectiveness and safety outcomes of real-world patients with AD using databases containing information on the stage of the disease, and cognitive, behavioral, and psychological assessment data are needed.

### Limitations

Our study is subject to the following main limitations. First, the study may be subject to selection bias owing to unobserved patient characteristics that are not commonly captured in Medicare claims, such as cognitive, functional, or neuropsychological assessment. Second, the mean (SD) follow-up period in our sample was short (1.5 [1.2] years); however, it is comparable to other prior observational studies.^27,30^ We could extend the follow-up period by relaxing our censoring criteria. Notwithstanding, we believe that these strict censoring criteria—which censor patients after a discontinuation or switch of the original treatment—strengthen our study by ensuring a clear attribution of outcome events to the treatment originally initiated after diagnosis of AD. Third, unfortunately, there is no evidence on the sensitivity and specificity of the use of claims data and *ICD-9* codes in the identification of some of the cardiovascular event outcomes included in our study. Potential low sensitivity and specificity would lead to misclassification of the outcome. However, this misclassification would likely be across all groups and should therefore not affect the internal validity of the study. Also, we did not restrict the number of days between diagnosis of AD and first antidementia medication prescription, which may have led to potential selection bias because differences in time to treatment initiation after diagnosis of AD could reflect different degrees of cognitive decline across groups.

## Conclusions

We found no differences in time to SNF admission across all antidementia medications available for the treatment of AD. However, memantine was associated with a lower risk of cardiovascular events compared with both AChEI monotherapy and combination therapy with an AChEI and memantine.

## Notes

Supplement. **eTable 1.** Unadjusted Cumulative Incidence of Outcome Events by Treatment Group **eTable 2.** Adjusted Hazard Ratios for Secondary Outcomes for the Comparison between Acetylcholinesterase Inhibitor Monotherapy, Memantine Monotherapy, and Combination Therapy **eTable 3.** Adjusted Hazard Ratios for Secondary Outcome by Treatment Group

## References

[1] McKhann GM, Knopman DS, Chertkow H, The diagnosis of dementia due to Alzheimer’s disease: recommendations from the National Institute on Aging–Alzheimer’s Association workgroups on diagnostic guidelines for Alzheimer’s disease. Alzheimers Dement. 2011;7(3):-. doi:10.1016/j.jalz.2011.03.005 21514250 PMC3312024

[2] Alzheimer’s Association 2018 Alzheimer’s disease facts and figures. Alzheimers Dement. 2018;14(3):367-429. doi:10.1016/j.jalz.2018.02.001 19426951

[3] Administration on Aging, Administration for Community Living, US Department of Health and Human Services A profile of older Americans: 2016 https://acl.gov/sites/default/files/Aging%20and%20Disability%20in%20America/2016-Profile.pdf. Accessed April 12, 2018.

[4] Birks JS, Grimley Evans J Rivastigmine for Alzheimer’s disease. Cochrane Database Syst Rev. 2015;(4):CD001191. 25858345 10.1002/14651858.CD001191.pub3

[5] Tan CC, Yu JT, Wang HF, Efficacy and safety of donepezil, galantamine, rivastigmine, and memantine for the treatment of Alzheimer’s disease: a systematic review and meta-analysis. J Alzheimers Dis. 2014;41(2):615-631. doi:10.3233/JAD-132690 24662102

[6] Di Santo SG, Prinelli F, Adorni F, Caltagirone C, Musicco M A meta-analysis of the efficacy of donepezil, rivastigmine, galantamine, and memantine in relation to severity of Alzheimer’s disease. J Alzheimers Dis. 2013;35(2):349-361. doi:10.3233/JAD-122140 23411693

[7] Tsoi KK, Chan JY, Chan FC, Hirai HW, Kwok TC, Wong SY Monotherapy is good enough for patients with mild-to-moderate Alzheimer’s disease: a network meta-analysis of 76 randomized controlled trials. Clin Pharmacol Ther. 2019;105(1):121-130. doi:10.1002/cpt.1104 29717478

[8] Birks J Cholinesterase inhibitors for Alzheimer’s disease. Cochrane Database Syst Rev. 2006;(1):CD005593. 16437532 10.1002/14651858.CD005593 PMC9006343

[9] Raina P, Santaguida P, Ismaila A, Effectiveness of cholinesterase inhibitors and memantine for treating dementia: evidence review for a clinical practice guideline. Ann Intern Med. 2008;148(5):379-397. doi:10.7326/0003-4819-148-5-200803040-00009 18316756

[10] Deardorff WJ, Grossberg GT A fixed-dose combination of memantine extended-release and donepezil in the treatment of moderate-to-severe Alzheimer’s disease. Drug Des Dev Ther. 2016;10:3267-3279. doi:10.2147/DDDT.S86463 27757016 PMC5055113

[11] Howard R, McShane R, Lindesay J, Donepezil and memantine for moderate-to-severe Alzheimer’s disease. N Engl J Med. 2012;366(10):893-903. doi:10.1056/NEJMoa1106668 22397651

[12] Tariot PN, Farlow MR, Grossberg GT, Graham SM, McDonald S, Gergel I; Memantine Study Group Memantine treatment in patients with moderate to severe Alzheimer disease already receiving donepezil: a randomized controlled trial. JAMA. 2004;291(3):317-324. doi:10.1001/jama.291.3.317 14734594

[13] Araki T, Wake R, Miyaoka T, The effects of combine treatment of memantine and donepezil on Alzheimer’s disease patients and its relationship with cerebral blood flow in the prefrontal area. Int J Geriatr Psychiatry. 2014;29(9):881-889. doi:10.1002/gps.4074 24436135

[14] Grossberg GT, Manes F, Allegri RF, The safety, tolerability, and efficacy of once-daily memantine (28 mg): a multinational, randomized, double-blind, placebo-controlled trial in patients with moderate-to-severe Alzheimer’s disease taking cholinesterase inhibitors. CNS Drugs. 2013;27(6):469-478. doi:10.1007/s40263-013-0077-7 23733403 PMC3680656

[15] Chen R, Chan PT, Chu H, Treatment effects between monotherapy of donepezil versus combination with memantine for Alzheimer disease: a meta-analysis. PLoS One. 2017;12(8):e0183586. doi:10.1371/journal.pone.0183586 28827830 PMC5565113

[16] Schmidt R, Hofer E, Bouwman FH, EFNS-ENS/EAN Guideline on concomitant use of cholinesterase inhibitors and memantine in moderate to severe Alzheimer’s disease. Eur J Neurol. 2015;22(6):889-898. doi:10.1111/ene.12707 25808982

[17] Howes LG Cardiovascular effects of drugs used to treat Alzheimer’s disease. Drug Saf. 2014;37(6):391-395. doi:10.1007/s40264-014-0161-z 24777654

[18] Winblad B, Gauthier S, Scinto L, ; GAL-INT-11/18 Study Group Safety and efficacy of galantamine in subjects with mild cognitive impairment. Neurology. 2008;70(22):2024-2035. doi:10.1212/01.wnl.0000303815.69777.26 18322263

[19] Hernandez RK, Farwell W, Cantor MD, Lawler EV Cholinesterase inhibitors and incidence of bradycardia in patients with dementia in the Veterans Affairs New England healthcare system. J Am Geriatr Soc. 2009;57(11):1997-2003. doi:10.1111/j.1532-5415.2009.02488.x 19793162

[20] Park-Wyllie LY, Mamdani MM, Li P, Gill SS, Laupacis A, Juurlink DN Cholinesterase inhibitors and hospitalization for bradycardia: a population-based study. PLoS Med. 2009;6(9):e1000157. doi:10.1371/journal.pmed.1000157 19787032 PMC2742897

[21] Gill SS, Anderson GM, Fischer HD, Syncope and its consequences in patients with dementia receiving cholinesterase inhibitors: a population-based cohort study. Arch Intern Med. 2009;169(9):867-873. doi:10.1001/archinternmed.2009.43 19433698

[22] Kim DH, Brown RT, Ding EL, Kiel DP, Berry SD Dementia medications and risk of falls, syncope, and related adverse events: meta-analysis of randomized controlled trials. J Am Geriatr Soc. 2011;59(6):1019-1031. doi:10.1111/j.1532-5415.2011.03450.x 21649634 PMC3260523

[23] Takaya T, Okamoto M, Yodoi K, Torsades de pointes with QT prolongation related to donepezil use. J Cardiol. 2009;54(3):507-511. doi:10.1016/j.jjcc.2009.03.011 19944332

[24] Fisher AA, Davis MW Prolonged QT interval, syncope, and delirium with galantamine. Ann Pharmacother. 2008;42(2):278-283. doi:10.1345/aph.1K514 18182475

[25] Poluzzi E, Raschi E, Moretti U, De Ponti F Drug-induced torsades de pointes: data mining of the public version of the FDA Adverse Event Reporting System (AERS). Pharmacoepidemiol Drug Saf. 2009;18(6):512-518. doi:10.1002/pds.1746 19358226

[26] Leitch A, McGinness P, Wallbridge D Calculate the QT interval in patients taking drugs for dementia. BMJ. 2007;335(7619):557. doi:10.1136/bmj.39020.710602.47 17855324 PMC1976518

[27] Fosbøl EL, Peterson ED, Holm E, Comparative cardiovascular safety of dementia medications: a cross-national study. J Am Geriatr Soc. 2012;60(12):2283-2289. doi:10.1111/j.1532-5415.2012.04241.x 23176182

[28] Gallini A, Sommet A, Montastruc JL; French PharmacoVigilance Network Does memantine induce bradycardia? a study in the French PharmacoVigilance Database. Pharmacoepidemiol Drug Saf. 2008;17(9):877-881. doi:10.1002/pds.1620 18500725

[29] Center for Medicare and Medicaid Services Chronic Conditions Data Warehouse Condition categories. https://www.ccwdata.org/web/guest/condition-categories. Accessed April 26, 2018.

[30] Bent-Ennakhil N, Coste F, Xie L, A real-world analysis of treatment patterns for cholinesterase inhibitors and memantine among newly-diagnosed Alzheimer’s disease patients. Neurol Ther. 2017;6(1):131-144. doi:10.1007/s40120-017-0067-7 28508250 PMC5447560

[31] von Elm E, Altman DG, Egger M, Pocock SJ, Gøtzsche PC, Vandenbroucke JP; STROBE Initiative The Strengthening the Reporting of Observational Studies in Epidemiology (STROBE) statement: guidelines for reporting observational studies. Lancet. 2007;370(9596):1453-1457. doi:10.1016/S0140-6736(07)61602-X 18064739

[32] Lopez OL, Becker JT, Wahed AS, Long-term effects of the concomitant use of memantine with cholinesterase inhibition in Alzheimer disease. J Neurol Neurosurg Psychiatry. 2009;80(6):600-607. doi:10.1136/jnnp.2008.158964 19204022 PMC2823571

[33] Howard R, McShane R, Lindesay J, Nursing home placement in the Donepezil and Memantine in Moderate to Severe Alzheimer’s Disease (DOMINO-AD) trial: secondary and post-hoc analyses. Lancet Neurol. 2015;14(12):1171-1181. doi:10.1016/S1474-4422(15)00258-6 26515660

[34] Gonzalez L A focus on the Program of All-Inclusive Care for the Elderly (PACE). J Aging Soc Policy. 2017;29(5):475-490. doi:10.1080/08959420.2017.1281092 28085633

