# Supplementary material for: Association of Antidementia Therapies With Time to Skilled Nursing Facility Admission and Cardiovascular Events Among Elderly Adults With Alzheimer Disease
Source: JAMA Netw Open. 2019 Mar 1;2(3):e190213. doi: 10.1001/jamanetworkopen.2019.0213 (PMC6484658; doi:10.1001/jamanetworkopen.2019.0213)
Supplement: Supplement. — eTable 1. Unadjusted Cumulative Incidence of Outcome Events by Treatment Group eTable 2. Adjusted Hazard Ratios for Secondary Outcomes for the Comparison between Acetylcholinesterase Inhibitor Monotherapy, Memantine Monotherapy, and Combination Therapy eTable 3. Adjusted Hazard Ratios for Secondary Outcome by Treatment Group [file jamanetwopen-2-e190213-s001.pdf]

Supplementary Online Content

San-Juan-Rodriguez A, Zhang Y, He M, Hernandez I. Association of antidementia therapies with time to skilled nursing facility admission and cardiovascular events among elderly adults with Alzheimer disease. *JAMA Netw Open.* 2019;2(3):e190213. doi:10.1001/jamanetworkopen.2019.0213

eTable 1. Unadjusted Cumulative Incidence of Outcome Events by Treatment Group
eTable 2. Adjusted Hazard Ratios for Secondary Outcomes for the Comparison between Acetylcholinesterase Inhibitor Monotherapy, Memantine Monotherapy, and Combination Therapy
eTable 3. Adjusted Hazard Ratios for Secondary Outcome by Treatment Group

This supplementary material has been provided by the authors to give readers additional information about their work.

© 2019 San-Juan-Rodriguez A et al. *JAMA Network Open.*

eTable 1. Unadjusted Cumulative Incidence of Outcome Events by Treatment Group

Cumulative incidence (95% CI)	Donepezil (n=36,463)	Galantamine (n=1,011)	Rivastigmine (n=6,950)	Memantine (n=3,362)	Combination therapy (n=17,242)
Effectiveness outcomes					
Skilled nursing facility admission					
At 1 year	0.209 (0.204 , 0.214)	0.189 (0.161 , 0.217)	0.213 (0.201 , 0.224)	0.220 (0.212 , 0.229)	0.204 (0.197 , 0.211)
At 2 years	0.325 (0.318 , 0.331)	0.293 (0.256 , 0.330)	0.324 (0.308 , 0.340)	0.341 (0.329 , 0.353)	0.322 (0.314 , 0.331)
At 3 years	0.412 (0.404 , 0.420)	0.385 (0.337 , 0.433)	0.415 (0.395 , 0.435)	0.427 (0.413 , 0.442)	0.416 (0.405 , 0.426)
Safety outcomes					
Any cardiovascular adverse event					
At 1 year	0.19 (0.19 , 0.20)	0.17 (0.15 , 0.20)	0.19 (0.17 , 0.20)	0.18 (0.17 , 0.18)	0.18 (0.17 , 0.18)
At 2 years	0.29 (0.28 , 0.29)	0.28 (0.24 , 0.32)	0.29 (0.27 , 0.30)	0.27 (0.26 , 0.29)	0.28 (0.27 , 0.29)
At 3 years	0.36 (0.36 , 0.37)	0.38 (0.33 , 0.43)	0.36 (0.34 , 0.38)	0.35 (0.33 , 0.36)	0.35 (0.34 , 0.36)
Acute myocardial infarction					
At 1 year	0.03 (0.03 , 0.03)	0.02 (0.01 , 0.03)	0.03 (0.03 , 0.04)	0.03 (0.03 , 0.03)	0.02 (0.02 , 0.03)
At 2 years	0.05 (0.05 , 0.06)	0.04 (0.02 , 0.05)	0.06 (0.05 , 0.07)	0.05 (0.05 , 0.06)	0.04 (0.04 , 0.05)
At 3 years	0.07 (0.07 , 0.08)	0.08 (0.05 , 0.11)	0.08 (0.07 , 0.09)	0.07 (0.06 , 0.08)	0.06 (0.05 , 0.06)
Atrioventricular block					
At 1 year	0.011 (0.009 , 0.012)	0.007 (0.001 , 0.013)	0.009 (0.006 , 0.012)	0.009 (0.007 , 0.011)	0.008 (0.006 , 0.009)
At 2 years	0.016 (0.014 , 0.018)	0.009 (0.001 , 0.018)	0.013 (0.009 , 0.017)	0.013 (0.010 , 0.016)	0.011 (0.009 , 0.013)
At 3 years	0.019 (0.017 , 0.022)	0.009 (0.001 , 0.018)	0.017 (0.012 , 0.022)	0.018 (0.014 , 0.022)	0.015 (0.012 , 0.017)
Bradycardia					
At 1 year	0.07 (0.07 , 0.08)	0.06 (0.04 , 0.08)	0.07 (0.06 , 0.08)	0.06 (0.06 , 0.07)	0.07 (0.06 , 0.07)
At 2 years	0.12 (0.11 , 0.12)	0.12 (0.09 , 0.14)	0.11 (0.10 , 0.12)	0.11 (0.10 , 0.12)	0.11 (0.11 , 0.12)

© 2019 San-Juan-Rodriguez A et al. *JAMA Network Open.*

At 3 years	0.16 (0.15 , 0.16)	0.18 (0.14 , 0.22)	0.14 (0.13 , 0.16)	0.14 (0.13 , 0.15)	0.15 (0.14 , 0.16)
QT prolongation					
At 1 year	0.001 (0.000 , 0.001)	0.002 (0.000 , 0.004)	0.000 (0.000 , 0.000)	0.001 (0.001 , 0.002)	0.001 (0.000 , 0.001)
At 2 years	0.001 (0.001 , 0.002)	0.003 (0.000 , 0.008)	0.000 (0.000 , 0.000)	0.003 (0.001 , 0.004)	0.001 (0.001 , 0.002)
At 3 years	0.002 (0.001 , 0.003)	0.003 (0.000 , 0.008)	0.000 (0.000 , 0.000)	0.003 (0.001 , 0.004)	0.002 (0.001 , 0.003)
Syncope					
At 1 year	0.11 (0.10 , 0.11)	0.11 (0.08 , 0.13)	0.10 (0.09 , 0.11)	0.10 (0.09 , 0.10)	0.10 (0.10 , 0.11)
At 2 years	0.17 (0.16 , 0.17)	0.17 (0.14 , 0.21)	0.16 (0.15 , 0.18)	0.15 (0.15 , 0.16)	0.17 (0.16 , 0.18)
At 3 years	0.21 (0.21 , 0.22)	0.21 (0.17 , 0.25)	0.21 (0.19 , 0.23)	0.20 (0.19 , 0.21)	0.22 (0.21 , 0.23)
Ventricular tachycardia					
At 1 year	0.011 (0.010 , 0.012)	0.011 (0.004 , 0.018)	0.013 (0.010 , 0.017)	0.011 (0.008 , 0.013)	0.009 (0.007 , 0.010)
At 2 years	0.017 (0.015 , 0.018)	0.019 (0.007 , 0.031)	0.022 (0.017 , 0.027)	0.016 (0.013 , 0.019)	0.013 (0.011 , 0.016)
At 3 years	0.022 (0.020 , 0.024)	0.027 (0.011 , 0.042)	0.025 (0.019 , 0.031)	0.021 (0.017 , 0.026)	0.018 (0.015 , 0.021)

NOTES:

Incidence rates were estimated from Kaplan Meier time-to-event curves.

© 2019 San-Juan-Rodriguez A et al. *JAMA Network Open.*

eTable 2. Adjusted Hazard Ratios for Secondary Outcomes for the Comparison between Acetylcholinesterase Inhibitor Monotherapy, Memantine Monotherapy, and Combination Therapy

	**Acute myocardial infarction**	**Atrioventricular block**	**Bradycardia**	**Syncope**	**QT prolongation**	**Ventricular tachycardia**
Memantine vs. AChEI	0.99 (0.89 , 1.09)	0.89 (0.73 , 1.08)	0.88 (0.82 , 0.95)**	0.92 (0.86 , 0.97)**	1.50 (0.84 , 2.65)	1.01 (0.84 , 1.22)
Combination vs. AChEI	0.88 (0.80 , 0.96)**	0.80 (0.67 , 0.95)**	0.99 (0.94 , 1.05)	1.04 (0.99 , 1.09)	1.04 (0.60 , 1.78)	0.95 (0.81 , 1.11)
Combination vs. Memantine	0.89 (0.79 , 1.01)	0.90 (0.71 , 1.14)	1.12 (1.03 , 1.22)**	1.14 (1.06 , 1.21)**	0.69 (0.36 , 1.35)	0.93 (0.75 , 1.15)

NOTES:

Abbreviations: AChEI, acetylcholinesterase inhibitor.

**p-value<.016 (Bonferroni corrected)

Adjusted hazard ratios were obtained from Cox proportional hazard models that controlled for age, gender, race, disability, history of acute myocardial infarction, history of bradycardia, history of syncope, history of atrioventricular block, history of QT interval prolongation, history of ventricular tachycardia, and each of 25 CMS priority conditions (all 27 CMS priority conditions except for AD and AD or other dementia).[29]

© 2019 San-Juan-Rodriguez A et al. *JAMA Network Open.*

eTable 3. Adjusted Hazard Ratios for Secondary Outcome by Treatment Group

	Acute myocardial infarction	Atrioventricular block	Bradycardia	Syncope	QT prolongation	Ventricular tachycardia
Galantamine vs. Donepezil	0.93 (0.66 , 1.32)	0.54 (0.24 , 1.21)	1.01 (0.82 , 1.24)	1.01 (0.85 , 1.210)	1.72 (0.41 , 7.21)	1.21 (0.71 , 2.07)
Rivastigmine vs. Donepezil	1.02 (0.89 , 1.16)	0.75 (0.58 , 0.98)*	0.88 (0.81 , 0.97)*	0.93 (0.86 , 1.00)*	0.14 (0.02 , 1.05)	1.15 (0.92 , 1.43)
Memantine vs. Donepezil	0.99 (0.89 , 1.10)	0.84 (0.69 , 1.03)	0.87 (0.81 , 0.94)**	0.91 (0.85 , 0.96)**	1.34 (0.75 , 2.39)	1.04 (0.86 , 1.25)
Combination vs. Donepezil	0.88 (0.80 , 0.96)*	0.76 (0.64 , 0.91)**	0.98 (0.92 , 1.03)	1.03 (0.98 , 1.08)	0.93 (0.54 , 1.60)	0.97 (0.82 , 1.14)
Rivastigmine vs. Galantamine	1.10 (0.76 , 1.58)	1.40 (0.60 , 3.25)	0.88 (0.70 , 1.09)	0.92 (0.76 , 1.11)	0.08 (0.01 , 0.93)*	0.95 (0.54 , 1.67)
Memantine vs. Galantamine	1.06 (0.75 , 1.52)	1.57 (0.69 , 3.58)	0.86 (0.69 , 1.07)	0.90 (0.75 , 1.08)	0.78 (0.18, 3.42)	0.86 (0.49 , 1.49)
Combination vs. Galantamine	0.95 (0.67 , 1.35)	1.41 (0.62 , 3.20)	0.96 (0.78 , 1.19)	1.02 (0.85 , 1.22)	0.54 (0.12 , 2.34)	0.80 (0.46 , 1.38)
Memantine vs. Rivastigmine	0.97 (0.83 , 1.13)	1.12 (0.83 , 1.52)	0.98 (0.88 , 1.10)	0.98 (0.89 , 1.07)	9.31 (1.23, 70.54)*	0.91 (0.70 , 1.18)
Combination vs. Rivastigmine	0.86 (0.75 , 1.00)	1.01 (0.76 , 1.34)	1.10 (1.00 , 1.22)	1.11 (1.02 , 1.20)*	6.45 (0.86 , 48.26)	0.85 (0.66 , 1.08)
Combination vs. Memantine	0.89 (0.79 , 1.01)	0.90 (0.71 , 1.14)	1.12 (1.03 , 1.22)*	1.14 (1.06 , 1.21)**	0.69 (0.36 , 1.35)	0.93 (0.75 , 1.15)

NOTES:

*p-value<.05; **p-value<.005 (Bonferroni corrected)

Adjusted hazard ratios were obtained from Cox proportional hazard models that controlled for age, gender, race, disability, history of acute myocardial infarction, history of bradycardia, history of syncope, history of atrioventricular block, history of QT interval prolongation, history of ventricular tachycardia, and each of 25 CMS priority conditions (all 27 CMS priority conditions except for AD and AD or other dementia).[29]

© 2019 San-Juan-Rodriguez A et al. *JAMA Network Open.*
